# Tuning protein half-life in mouse using sequence-defined biopolymers functionalized with lipids

**DOI:** 10.1073/pnas.2103099119

**Published:** 2022-01-18

**Authors:** Koen Vanderschuren, Pol Arranz-Gibert, Minsoo Khang, Dagan Hadar, Alice Gaudin, Fan Yang, Ewa Folta-Stogniew, W. Mark Saltzman, Miriam Amiram, Farren J. Isaacs

**Affiliations:** ^a^Department of Molecular, Cellular & Developmental Biology, Yale University, New Haven, CT 06520;; ^b^Systems Biology Institute, Yale University, West Haven, CT 06516;; ^c^Department of Biomedical Engineering, Yale University, New Haven, CT 06520;; ^d^Avram and Stella Goldstein-Goren Department of Biotechnology Engineering, Ben-Gurion University of the Negev, Beer-Sheva 84105, Israel;; ^e^W. M. Keck Biotechnology Research Laboratory, Yale University School of Medicine, New Haven, CT 06511;; ^f^Department of Chemical & Environmental Engineering, Yale University, New Haven, CT 06520;; ^g^Department of Cellular & Molecular Physiology, Yale University, New Haven, CT 06511

**Keywords:** synthetic biology, protein engineering, noncanonical amino acids, serum protein half-life extension, genome recoding

## Abstract

The use of biologics in the treatment of numerous diseases has increased steadily over the past decade due to their high specificities, low toxicity, and limited side effects. Despite this success, peptide- and protein-based drugs are limited by short half-lives and immunogenicity. To address these challenges, we use a genomically recoded organism to produce genetically encoded elastin-like polypeptide–protein fusions containing multiple instances of *para*-azidophenylalanine (pAzF). Precise lipidation of these pAzF residues generated a set of sequence-defined synthetic biopolymers with programmable binding affinity to albumin without ablating the activity of model fusion proteins, and with tunable blood serum half-lives spanning 5 to 94% of albumin’s half-life in a mouse model. Our findings present a proof of concept for the use of genetically encoded bioorthogonal conjugation sites for multisite lipidation to tune protein stability in mouse serum. This work establishes a programmable approach to extend and tune the half-life of protein or peptide therapeutics and a technical foundation to produce functionalized biopolymers endowed with programmable chemical and biophysical properties with broad applications in medicine, materials science, and biotechnology.

## Significance

Functionalization of proteins and biopolymers with chemical modifications can be utilized to alter their chemical and biophysical properties. In contrast to traditional chemical functionalization strategies, the use of nonstandard amino acids enables precise positioning of functional groups. Here, we report that multisite conjugation of fatty acids, at precise sites harboring genetically encoded nonstandard amino acids with bioorthogonal chemical handles, can be employed to tune the half-life of proteins in a mouse model. This programmable approach could offer a technical foundation for the modification of protein and peptide therapeutics to improve their efficacy or pharmacokinetic profile (e.g., to prevent rapid clearance and reduce frequency of administration).

A major goal of synthetic biology is to harness biological systems to produce valuable products, such as new therapeutics, renewable chemicals, and functionalized materials. In the case of proteins, the native translation process uses information encoded in DNA to guide their template-directed production at monomeric precision, albeit limited to the chemistry of the 20 natural amino acids. Work in genetic code expansion with nonstandard amino acids (nsAAs) has expanded the chemical palette of biology through the template-directed biosynthesis of proteins with synthetic chemistries (1). To date, such work has been limited to only one or a few instances of site-specific incorporation of nsAAs per protein, constraining biopolymer synthesis to tag-and-modify approaches or simple protein decorations. Recent advances include an increase in the number and chemical diversity of nsAAs (1), the development of highly active translation machinery for efficient incorporation of nsAAs into proteins (2, 3), and engineered strains of *Escherichia coli* with recoded genomes possessing open coding channels that can be dedicated to the incorporation of nsAAs (4–6). Together, these advances enable multisite incorporation of nsAAs to endow proteins and sequence-defined biopolymers with new chemical and biophysical properties.

An active area of interest for the use of nsAAs is to enhance the functionality of protein and peptide pharmaceuticals. They represent a versatile and fast-growing class of biological therapeutics (7, 8) that are particularly attractive as potential pharmaceuticals due to their high specificity, high activity and, in the case of peptides, rapid tissue penetration (7). However, major barriers prevent the widespread clinical use of many peptide or protein-based therapeutics (7): 1) the need to administer them by injection, 2) their rapid clearance by the kidneys, and 3) their rapid proteolytic degradation. As a result, these pharmaceuticals must be frequently administered at high doses, leading to a “peak-and-valley” pharmacokinetic profile. These characteristics can negatively affect therapeutic efficacy, can cause undesirable side effects with reduced patient compliance (9–12), and can trigger an immune response, including the induction of a neutralizing antibody response (13, 14).

To address these challenges, proteins and peptides are frequently functionalized to extend their half-life and improve immunotolerance. A widely adopted strategy is the conjugation of poly(ethylene glycol) (PEG), which increases the radius of the protein and reduces proteolytic cleavage, and consequently reduces clearance (15). However, the development of alternatives to PEGylation remains important, as PEGylation does not always offer the desired effect on pharmacokinetics, and in certain cases, safety concerns about its immunogenicity and accumulation in tissues have been raised (16–18). An alternative strategy is to conjugate or fuse the therapeutic protein or peptide to serum proteins with long half-lives, such as serum albumin, antibodies (e.g., full-length or fragments of IgG), or blood components, such as red blood cells (17, 18). Similarly, many approaches use chemical moieties or peptides to promote noncovalent binding interactions to the same serum proteins and complexes in order to extend half-life (17–20). One effective and safe option is the use of fatty acids (FAs) to promote binding to serum albumin. For example, insulin and glucagon-like peptide-1 (GLP-1) conjugated with a single FA are clinically used to treat diabetic patients (21–23).

A major hurdle to the development of functionalized therapeutics is to selectively and predictably modify the protein while maintaining bioactivity. Conventional strategies for PEGylation and functionalization with chemical moieties utilize chemistries that modify the target protein at their termini, or at residues with reactive side-chains (24). The functionalization at C or N termini can be highly selective and predictable, but it can reduce bioactivity and is thus incompatible with many proteins. In contrast, modifications at reactive side-chains (e.g., cysteine or lysine) is less restrictive, but it can be difficult (or practically impossible) to identify unique reactive sites in the peptide sequence for site-specific conjugation. To address this problem, amino acids in the protein are typically mutagenized, which can result in reduced bioactivity. Recently, nsAAs have been successfully employed for modification of proteins and peptides, offering bioorthogonal chemistries for functionalization at predetermined positions within the protein (24–26). For example, human growth hormone (hGH) and fibroblast growth factor 21 (FGF21) were site-specifically PEGylated, prolonging their function through extended serum half-life in clinical trials (27, 28). In other cases, site-specific lipidation at a single nsAA was shown to extend half-life in mouse models (29, 30). Although these approaches have improved protein half-life, typically these designs are constrained to a single instance of the nsAA, which limits the versatility and tunability (e.g., customized range of half-lives) of these functionalized peptides and proteins.

In this study, we present a synthetic biology platform to biosynthesize protein–polymer fusions with sequence-defined conjugation sites for multisite lipidation in order to extend and tailor the half-life of proteins in vivo. Specifically, we encoded up to 10 instances of the nsAA *para*-azidophenylalanine (pAzF) in elastin-like polypeptide (ELP) fusion proteins at high yields in an engineered bacterium with a recoded genome (4), and demonstrate the ability to precisely control the position and number of FAs per biopolymer. We found that the number of FAs per protein is strongly correlated with the binding affinity to serum albumin, enabling us to tune the in vivo serum half-life of proteins without accumulation in organs or eliciting an inflammatory response in mouse. These advances could be applied to extend and tune the half-life of protein or peptide therapeutics and establish a technical foundation to produce sequence-defined programmable biopolymers endowed with bespoke chemical and biophysical properties with broad applications in medicine, materials science, and biotechnology.

## Results

### Design of Sequence-Defined Synthetic Biopolymers.

**Fig. 1.**
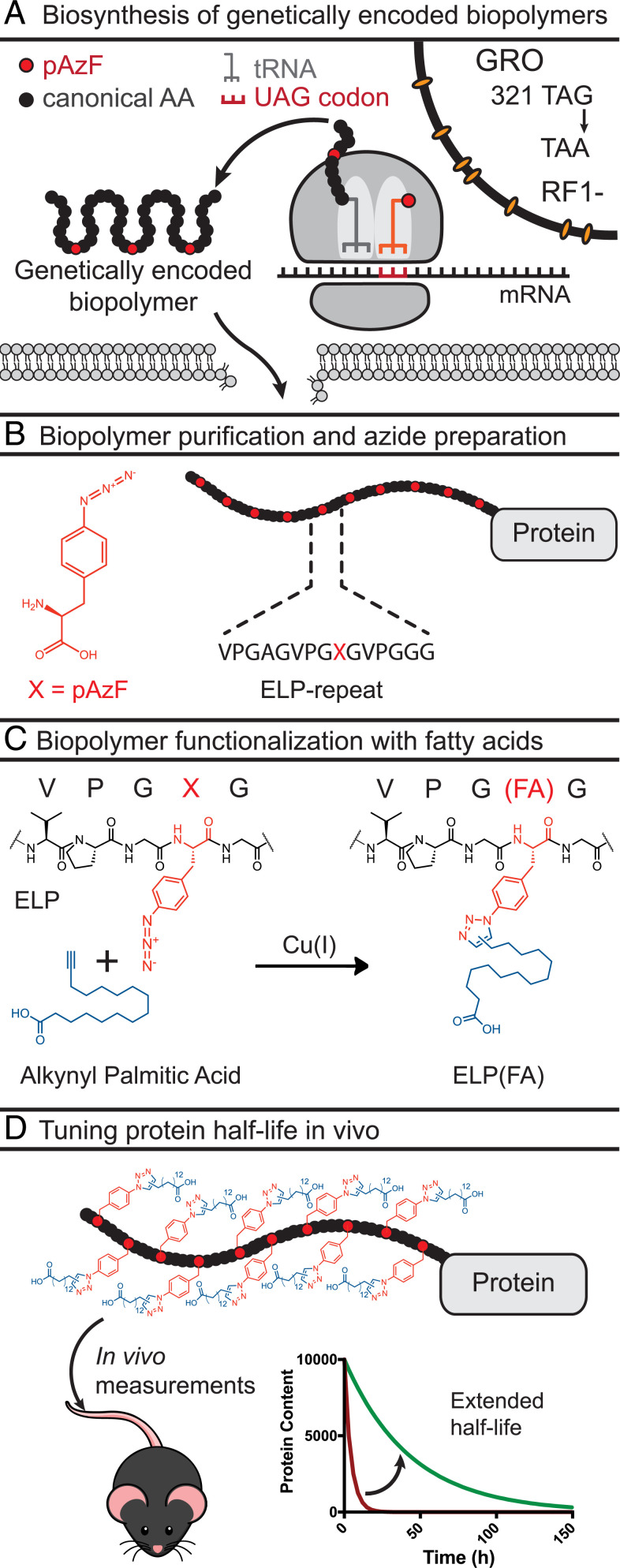
Biosynthesis and functionalization of genetically encoded biopolymers for half-life extension of proteins. (*A*) Site-specific multisite incorporation of pAzF at UAG codons in the GRO. All 321 TAG codons in *E. coli* were genomically recoded to TAA. To create the GRO, RF1 was deleted. The canonical amino acids and pAzF are shown as black and red circles, respectively. The TAG codon is converted into a sense codon for multisite incorporation of pAzF. (*B*) Schematic of the ELP-protein with 10 pAzF residues. The chemical structure of pAzF and the sequence of a single ELP repeat are shown. (*C*) Functionalization of azido groups in ELPs through copper(I)-mediated click chemistry with alkynyl palmitic acid. (*D*) Functionalized biopolymers are characterized in mice to study impact on half-life.

To enable the biosynthesis of sequence-defined synthetic biopolymers with template-directed conjugation sites, we utilized a recently described synthetic biology expression system that allows efficient incorporation of nsAAs (e.g., pAzF) at UAG codons (Fig. 1*A*) (2). This system possesses two unique properties. First, the expression host is the genomically recoded organism (GRO) (4), an *E. coli* MG1655 derivative, in which all instances of UAG stop codons were recoded to synonymous UAA codons, followed by the deletion of release factor 1 (RF1). This GRO establishes an open codon by eliminating competition between an orthogonal tRNA_CUA_/aminoacyl tRNA synthase (aaRS) pair and termination at UAG codons by RF1. Second, aaRSs evolved for aminoacylation with nsAAs typically have significantly reduced activities compared to native enzymes, resulting in low levels of nsAA-tRNA and low yields for proteins with multiple instances of an nsAA (31). Here, we use a tRNA_CUA_/aaRS derived from *Methanocaldococcus jannaschii* that was evolved for enhanced activity, enabling efficient multisite incorporation of nsAAs into proteins (2). Together, this expression system enables the biosynthesis of polypeptide polymers with multiple pAzF residues at high yields and accuracy.

To study the effect of the number of FA conjugates on the in vivo serum half-life, we chose to introduce the nsAA in an ELP with 10 consecutive pentadecapeptide repeats for functionalization. ELPs have previously been fused to active therapeutic peptides for a variety of indications, including type 2 diabetes and cancer (32), and serve as a versatile module to alter their pharmacokinetics. Within each repeat of the ELP, we encoded either a tyrosine or a pAzF residue at a designated guest residue position (henceforth named the “target residue”) (Fig. 1*B*), such that the genetic template controls the number and position of pAzF residues in the ELP-GFP. In turn, the bioorthogonal copper(I)-catalyzed azide-alkyne Huisgen cycloaddition (click-chemistry) reaction between pAzF residues and palmitic acid alkynes (with an ω terminal alkyne, such that the carboxyl group is exposed) ensures site-specific conjugation (Fig. 1 *C* and *D*). Alkynyl palmitic acid was used for functionalization because it had previously been shown to strongly promote binding to albumin (23).

### Lipidation at Genetically Encoded pAzF Residues.

**Fig. 2.**
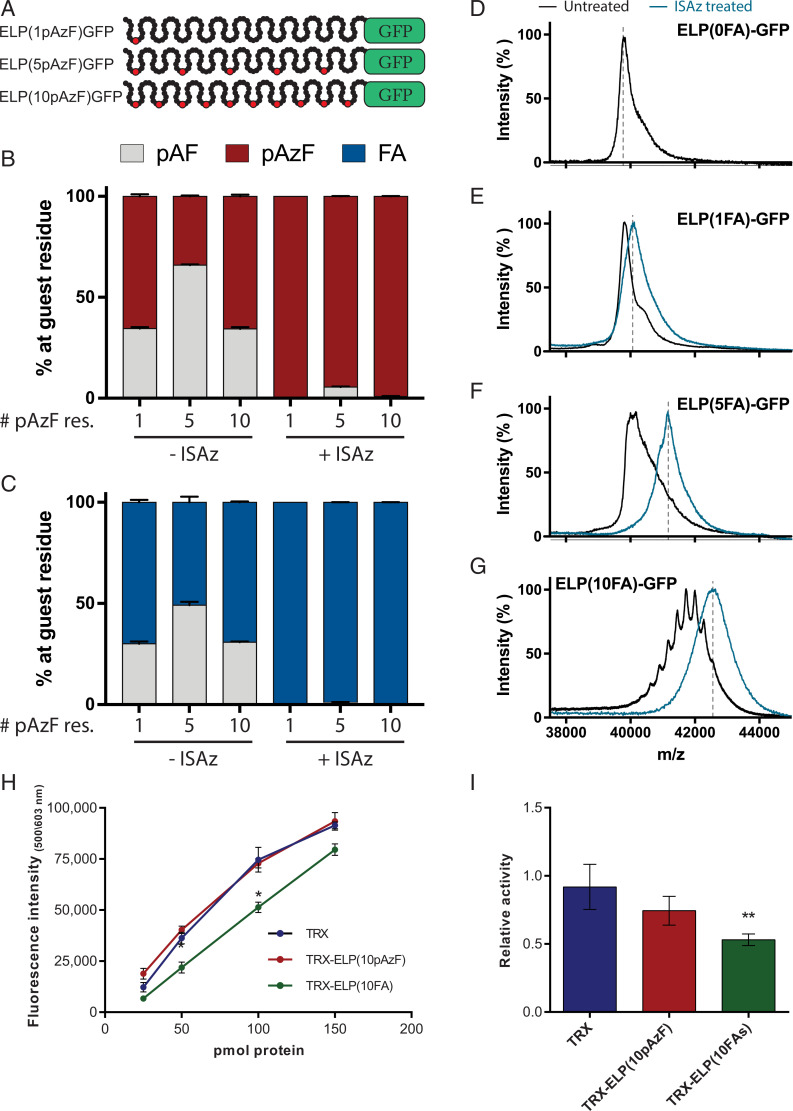
Analysis of the purity and bioactivity of multisite lipidated biopolymers. (*A*) Schematic representation of ELP-GFP reporter constructs with 1, 5, or 10 pAzF residues. Target positions for pAzF are indicated in red. (*B*) Relative abundance of detected nonstandard amino acids at target residues of ELP units based on quantitative MS. Treatment with ISAz selectively converts reduced pAzF residues, pAF, back into pAzF. (*C*) Click-chemistry with FA alkynes functionalizes all pAzF, but not pAF, residues (*n* = 3, error bars: mean ± SD). (*D–G*) Intact MS of full-length ELP(FA)-GFP after click-chemistry with (blue) or without (black) ISAz treatment. (*H*) Activity of recombinant trx, trx-ELP(10pAzF), and trx-ELP(10FA) at protein quantities ranging from 25 to 150 pmol per well. (*I*) Effect of HSA on the activity of recombinant trx, trx-ELP(10pAzF) and trx-ELP(10FA) using 100 pmol of each protein and 500 pmol HSA. Data are normalized to the activity of each protein without HSA (*n* = 3, error bars: mean ± SEM). *P < 0.05, **P < 0.01.

To evaluate if we could produce proteins with a genetically controlled number of FAs, we expressed ELP-GFP with 0, 1, 5, or 10 UAG codons at a yield of ∼70 mg/L (Fig. 2*A* and *SI Appendix*, Fig. S1). To carefully examine the fidelity and efficiency of each step in our system, we performed quantitative mass spectrometry (MS) analysis of the ELPs digested with thermolysin, which liberates each of the 10 constituent ELP units. To account for differences in ionization efficiency between the different peptide species, ion counts were quantified using a standard curve for each peptide (*SI Appendix*, Fig. S2). We first evaluated the efficiency of pAzF incorporation and found that the abundance of ELP units with pAzF was directly proportional to the number of UAG codons in the construct (*SI Appendix*, Fig. S3). Consistent with prior work (2), when all 10 ELP units contained a UAG codon, we detected minor (<5%) tyrosine misincorporation.

While examining the fidelity of pAzF incorporation, we observed significant levels of *para-*aminophenylalanine (pAF) (Fig. 2*B*), the reduced form of pAzF, which cannot participate in click-chemistry. In our system, pAF is the result of pAzF reduction (2, 33), and causes significant impurities and heterogeneity in the final preparations if left unresolved. To overcome this impurity, we developed a method to selectively recover pAzF from pAF with the diazotransfer reagent imidazole-1-sulfonyl azide (ISAz) (*Materials and Methods*) (34). We previously demonstrated that this approach enabled effective conversion of pAF residues to pAzF, without introducing azides at other primary amines found in ELP-GFPs (34). Consistent with this prior work, after treatment with ISAz we observed less than 5% of pAF via quantitative MS in each of the digested ELP-GFP constructs (Fig. 2*B*).

We then used click-chemistry to attach alkynyl palmitic acid at the precise positions where pAzF was encoded and assessed the purity of each ELP-GFP construct. These functionalized constructs are denoted as ELP(*n*FA)-GFP, where *n* indicates the number of UAG codons encoding pAzF in the template. We observed that all pAzF residues were converted to FA conjugates and no further reduction to pAF was detected during this reaction (Fig. 2*C*), emphasizing the high efficiency of this conjugation strategy. To complement the quantification at the peptide level, we used MS of the intact protein to evaluate the purity of the products (*Materials and Methods*). We consistently observed one dominant peak at the expected mass after ISAz treatment, whereas untreated samples demonstrated heterogeneous modification of the ELP-GFP (Fig. 2 *D*–*G*). For example, the ELP(10FA)-GFP without ISAz treatment showed multiple distinct peaks corresponding to an impure biopolymer with variable number of FAs. The peak profile correlates with a binomial distribution determined by the availability of pAzF residues (*SI Appendix*, Fig. S4) and suggests pAzF reduction is probabilistic.

Finally, we evaluated if the addition of multiple FAs per protein would impair solubility of the resulting constructs. The solubility of unmodified ELP-GFP and ISAz-treated proteins with 1, 5, or 10 FAs were determined by dynamic light scattering (DLS) analysis. All constructs, before and after FA conjugation, were soluble (>99% by volume) and did not self-assemble in solution (*SI Appendix*, Fig. S5). Together, these results demonstrate that the genetically controlled placement of pAzF and chemical regeneration of reduced pAF residues enable the programmable and robust functionalization of biopolymers at multiple sites.

### Functionalized Biopolymers Maintain Activity of Fusion Proteins.

We evaluated the effect of ELP and FA conjugation of peptide bioactivity on the activity of two proteins. We first quantified the effect of FA conjugation on GFP fluorescence, and found that it is reduced by ∼14 to 25% (*SI Appendix*, Fig. S6). In addition, to examine the effect of ELP fusion and FA conjugation on enzymatic activity, we produced and characterized thioredoxin (trx)-ELP fusion proteins. We evaluated the activity of recombinantly produced trx, trx-ELP(10pAzF), and trx-ELP(10FA), after ISAz treatment. Similar bioactivity was observed for trx and trx-ELP(10pAzF), while trx-ELP(10FA) retained greater than 50% bioactivity (Fig. 2*H*). In addition, we evaluated the effect of human serum albumin (HSA) binding on the activity of these proteins. To this end, we performed an activity assay in the presence of fivefold excess HSA concentration, and found that HSA binding reduced the activity of trx-ELP(10FA) by ∼50%, while activity of trx and trx-ELP(10pAzF) were not significantly reduced (Fig. 2*I*). Although FA conjugation and HSA binding may partially reduce bioactivity, these analyses demonstrate that posttranslational functionalization with FAs is compatible with bioactive proteins.

### Functionalized Biopolymers Exhibit Tunable Half-Life in Mice.

Since prior work with single FA conjugations of insulin showed that serum half-life is correlated with the binding affinity to albumin (23), we hypothesized that multisite lipidation of ELP-GFPs would significantly enhance binding affinity to mouse serum albumin (MSA), and consequently extend serum half-life, compared to a single conjugated FA. To study the impact of increasing the number of FAs, we analyzed ELP-GFP constructs (both with and without ISAz treatment) with surface plasmon resonance (SPR). The *K*_D_ values of our constructs were estimated based on the steady-state binding (Table 1). There was no detectable binding between MSA and the negative control without conjugated FAs [ELP(0FA)-GFP]. For untreated biopolymers, we found that the *K*_D_ with a single FA, ELP(1FA)-GFP (*K*_D_ = 126 ± 32 μM), was lowered 12- to 45-fold for ELP(5FA)-GFP (*K*_D_ = 10.4 ± 4.0 μM) and ELP(10FA)-GFP (*K*_D_ = 2.8 ± 0.2 μM), respectively. For the ISAz-treated biopolymers, we observed much stronger binding overall: treated ELP(1FA)-GFP presented a *K*_D_ of 25.9 ± 7.1 μM, and an increase to 5 and 10 FAs per protein further lowered the *K*_D_ to 4.0 ± 1.6 μM and 2.22 ± 0.03 μM, respectively. These data indicate that the affinity for MSA is strongly enhanced by conjugation of multiple FAs per protein and confirm that the binding affinity is correlated with the number of FAs.

**Table 1.** Binding affinity of ELP-GFP constructs for serum albumin

Construct	ISAz treated	*K*_D_ MSA (μM)	*K*_D_ HSA (μM)
ELP(0FA)-GFP	No	n.d.	n.d.
ELP(1FA)-GFP	No	126 ± 32.2	n.a.
ELP(5FA)-GFP	No	10.3 ± 4.0	n.a.
ELP(10FA)-GFP	No	2.76 ± 0.19	n.a.
ELP(1FA)-GFP	Yes	25.9 ± 7.1	19.3 ± 3.9
ELP(5FA)-GFP	Yes	4.0 ± 1.6	3.16 ± 0.60
ELP(10FA)-GFP	Yes	2.22 ± 0.03	1.64 ± 0.17

The *K*_D_ values were calculated from the steady-state affinity binding model obtained by SPR (*Materials and Methods*). Mean ± SD values were derived from four to eight independent experiments. n.d. indicates that no binding was detected and n.a. indicates not analyzed.

**Fig. 3.**
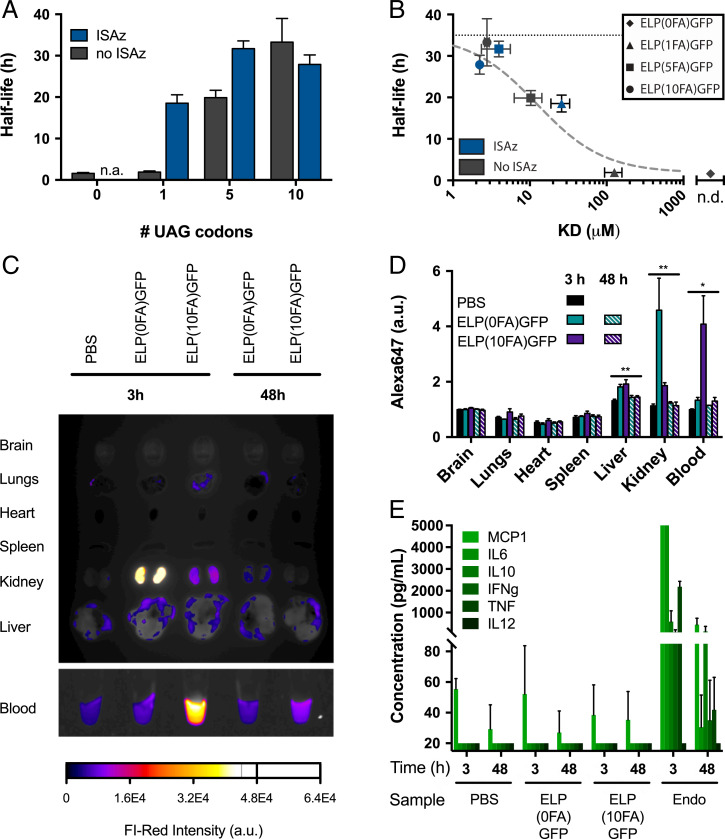
In vivo characterization of lipidated biopolymers in mouse. (*A*) Serum half-life measurements of lipidated biopolymers with or without treatment with ISAz. Measurements were collected after a single intravenous injection of 50 μg biopolymer in C57BL/6J mice. ELP(0FA)-GFP after ISAz treatment was not measured (*n* = 4, error bars: mean ± SD). (*B*) Correlation between the *K*_D_ and half-life of lipidated biopolymers with or without ISAz treatment. The horizontal, black dotted line shows the half-life of MSA, the dashed gray line shows model predictions (*n* = 4 to 8, error bars: mean ± SD, n.d. = not detected). (*C*) Distribution of biopolymers in mouse organs, 3 and 48 h after intravenous injection of Alexa Fluor 648–labeled ELP(0FA)-GFP or ELP(10FA)-GFP. The biopolymers were treated with ISAz. The data are representative of four independent measurements. (*D*) Quantification of average Alexa Fluor 648 intensity for organs shown in *C* (*n* = 4, error bars: mean ± SEM). For each organ separately, a one-way ANOVA was used to determine whether the differences between the means of the five treatment groups were statistically significant. After multiple testing correction, *P < 0.05 and **P < 0.005. (*E*) Serum concentration of select inflammatory cytokines at 3 and 48 h after injection. Endotoxin (100 µg) and PBS were used as positive and negative controls, respectively. Measurements below the lower limit of detection (20 pg/mL) or above the upper limit of detection (5,000 pg/mL) are plotted at their limit of detection (*n* = 3, error bars: mean ± SD).

We next determined if the tighter binding affinity is translated to prolonged half-life in C57BL/6J mice. A total of 50 μg of each protein variant (10 μM in phosphate-buffered saline [PBS]) was injected intravenously and blood was collected after 1, 4, 8, 16, and 24 h, followed by daily collections for 7 d. The blood levels of ELP(*n*FA)-GFP constructs were measured using a GFP-specific ELISA, and their pharmacokinetic profiles were calculated (*SI Appendix*, Fig. S7 and Table S1). We observed a striking 16- to 19-fold increase in half-life from 1.7 h for ELP(0FA)-GFP to 28 to 33 h for ISAz-treated ELP(5FA)-GFP and ELP(10FA)-GFP, as well as for untreated ELP(10FA)-GFP (Fig. 3*A*). Notably, when the same constructs were injected subcutaneously, we observed a delayed peak concentration, but the half-lives were equivalent to intravenous injections (*SI Appendix*, Figs. S8 and S9). Furthermore, these data show that the half-life of tight binding ELP-GFP constructs with multiple FA conjugates approaches the half-life of MSA in mice (35 h) (35), and is similar to the half-life of 28 h reported for protein–MSA fusion proteins (36).

Finally, we note that the observed *K*_D_ values can be approximated by dividing the *K*_D_ of ELP(1FA)-GFP (ISAz-treated) by the average number of conjugated FAs (*SI Appendix*, Fig. S10). This correlation has two key implications. First, biopolymers with high numbers of FA conjugates [i.e., ISAz-treated ELP(5FA)-GFP, and both treated and untreated ELP(10FA)-GFP] will have similar binding affinities to albumin, and therefore also similar half-lives. Second, tuning of the binding affinity and half-life is most pronounced at lower numbers of conjugated FAs. In the case of ISAz-treated ELP(10FA)-GFP, we found a small decrease (although not statistically significant) in half-life compared to treated ELP(5FA)-GFP and untreated ELP(10FA)-GFP. We hypothesize that denser packing of the 10 FAs does not improve, or may even reduce, the availability of FAs for albumin binding, highlighting the value of being able to precisely control the number of FAs per protein.

### Model of Lipidized Biopolymers Kinetics and Serum Half-Life.

We computationally modeled the system to gain a deeper understanding of the correlation between the binding affinity and half-life (*SI Appendix*, *Supplementary Information Text* and Fig. S11). In brief, a set of ordinary differential equations describes the binding and release of ELP-GFP from albumin as a function of the *K*_D_, as well as the clearance of both bound and unbound ELP-GFP. Here, unbound ELP-GFP has a half-life of 1.7 h, as empirically determined, and bound ELP-GFP is cleared at the same rate as albumin (35 h for mice) (35). Importantly, the half-life of the protein is determined by three parameters in this model: 1) the half-life of unbound protein, 2) the half-life of serum albumin, and 3) the binding affinity between the protein and albumin. By simulating the kinetics over time, we were able to calculate the overall clearance rate, and predictions made by the model were in good agreement with the empirical measurements for *K*_D_ and half-life (Fig. 3*B* and *SI Appendix*, Fig. S11). This suggests predictive capability for the half-life based on empirically determined *K*_D_ values, or the model can provide a target *K*_D_ based on the desired half-life. Together, our results confirm that titrating the number of FAs allows predictable tuning of the protein half-life by modifying the binding affinity to albumin.

### Functionalized Biopolymers Are Biocompatible and Noninflammatory.

To evaluate the biocompatibility of the ELP-GFP constructs, we assessed the biodistribution and inflammatory response in mouse. ELP(0FA)-GFP and ELP(10FA)-GFP labeled with Alexa Fluor 647 dye were administered intravenously, and after 3 or 48 h, the brain, lungs, heart, spleen, liver, kidneys, and blood were collected and imaged for far-red fluorescence (Fig. 3 *C* and *D* and *SI Appendix*, Fig. S12). In the case of ELP(0FA)-GFP, most of the reporter had cleared from the blood after 3 h, and a strong signal was observed in the kidney, whereas ELP(10FA)-GFP was clearly observed in the blood, and to a lesser extent in the kidney. For both samples, a small increase (20 to 30%) in signal was observed in the liver. After 48 h, the Alexa Fluor 647 signal was only observed in the blood of ELP(10FA)-GFP–injected mice, whereas the intensity in all other organs had returned to the basal level seen in the PBS injection control. These results are consistent with the rapid clearance of ELP(0FA)-GFP, which is likely to occur mostly through excretion from the kidneys. The blood from each of these conditions was further analyzed for signs of inflammation. We did not detect any elevation of proinflammatory cytokine levels after injection of ELP-GFP constructs compared to PBS injection, whereas injection with lipopolysaccharide (LPS) as positive control gave a clear inflammatory response at both 3 and 48 h (Fig. 3*E*). Together, these results show that FA conjugation enables half-life extension without long-term accumulation in organs, or eliciting an inflammatory response after intravenous injection.

Considering applications of this technology for peptide and protein drug delivery in humans, we evaluated if the use of multiple FAs per protein conveyed similar increases in binding affinity to HSA. We observed *K*_D_ values of 19.3 ± 3.9 μM, 3.2 ± 0.6 μM, and 1.6 ± 0.2 μM for ELP-GFP constructs with 1, 5, and 10 FAs per protein, respectively (Table 1). These binding affinities closely mirror the values observed for MSA, suggesting that multisite lipidation of proteins could be a promising strategy to tailor protein half-life in humans.

## Discussion

In this study, we describe the design and production of sequenced-defined synthetic biopolymers conjugated with a programmable number of FAs to tailor the serum half-life of proteins. Specifically, the genetically encoded pAzF residues facilitate precise and programmable functionalization with FAs, which enables titration of the binding affinity to both MSA and HSA. We determined that the binding affinity to albumin was predictive of the serum half-life in mice, suggesting that the protein clearance can be tuned by controlling the number of conjugated FAs per protein. Notably, we measured serum half-lives of up to 33 h, which is ∼94% of the 35-h half-life of MSA. Importantly, with similar binding affinities for MSA and HSA, we hypothesize that the half-life of these same constructs will be higher in humans, given that HSA has a significantly longer half-life (∼19 d) (37). Furthermore, activity analysis of a trx fusion protein, trx-ELP(10FA), indicate at most a 50% activity loss (in the absence or presence of HSA), as compared with free trx. This compares favorably with other carriers reported to cause an ∼30- to 500-fold reduction in the activity of other peptides (38–42). Although, as is true for any carrier, the effect of ELP fusion and FA conjugation on activity is expected to vary for each individual peptide, protein, or molecule, our proposed fusion partner is highly tunable, in ELP size, sequence, and FA number and position, which should enable future optimization of both pharmacokinetics and bioactivity of each drug candidate.

Lipidation is an appealing alternative to PEG, which has come under scrutiny due to concerns about immunogenicity (43, 44), and uncertainty about its degradation and clearance from the body (45). The use of FAs has clinical precedence, offers greater tunability than direct fusion to albumin, and has a well-established safety profile (46). However, the utility of current lipidation strategies is constrained by two factors. First, typically only moderate half-life extensions are achieved due to weak binding of pharmaceuticals with single FAs to albumin. Second, the ability to identify uniquely reactive residues without impacting bioactivity remains challenging with conventional labeling strategies. Our work addresses both limitations with a general methodology that enables tuning the half-life extension by titrating the number of FAs per protein, and the ability to design conjugation sites at monomeric precision enables facile screening of permissive residues to maintain bioactivity.

Unique to this work is the multisite and programmable placement of nsAAs to produce a biopolymer with tunable properties, enabled by sequence-defined insertion of multiple FAs per biopolymer for functionalization. The ELP can be placed at either of the termini, or pAzF residues can be positioned in the primary sequence of the protein, permitting the optimization of both bioactivity and half-life extension, which highlights the flexibility of this approach. Furthermore, bioorthogonal conjugation sites, such as pAzF residues, allow the attachment of a wide variety of chemical moieties to expand the palette of biological chemistry far beyond FAs at genetically encoded positions throughout the protein to enhance its functionality. This establishes a foundation for a new class of synthetic, sequence-defined biopolymers comprised of a combination of natural and synthetic monomers that unites the diversity of the chemical world with the monomeric precision of translation in biological systems. These biopolymers are uniquely enabled by recoded organisms with open coding channels dedicated to the template-directed incorporation of synthetic monomers. We envision that this work, together with further recoding efforts to open up additional coding channels dedicated for multiple distinct nsAAs (4–6), establishes the basis for novel and programmable biopolymers (47, 48) with broad utility in biological research, pharmaceuticals, materials science, and biotechnology.

## Materials and Methods

### Strains and Protein Expression.

All proteins were expressed in the GRO (*E. coli* C321.ΔA, CP006698.1, GI:54981157) (4) containing a previously described OTS plasmid –pAcFRS.1.t1– with a p15A origin of replication and chloramphenicol acetyltransferase selection marker (2). The ELP-GFP genes were expressed from a plasmid with colE1 origin of replication and a kanamycin resistance marker (*SI Appendix*, Fig. S13). Each ELP-GFP construct had 10 repetitive units of 15 amino acids (VPGAGVPGXGVPGGG), where residue X is either tyrosine or pAzF (*SI Appendix*, Tables S2 and S3). The gene for trx was chemically synthesized (IDT) and cloned using EcoRI and PpuMI restriction enzymes into an expression vector containing to create trx-ELP(10UAG) (*SI Appendix*, Table S4). The same trx gene was cloned into an empty-expression vector for expression of unfused trx.

All cultures were grown at 34 °C under shaking (220 rpm). Before expression, the expression strains were grown to confluence in 50 mL 2xYT media. This culture was used to inoculate 1 liter of 2xYT, containing 30 µg/mL chloramphenicol, 20 µg/mL kanamycin, 0.2% arabinose, and 1 mM pAzF. After 4 h, expression of ELP-GFP was induced, using a final concentration of 60 ng/mL anhydrotetracycline. Cells were harvested 24 h after inoculation by centrifugation at 4,000 × *g* for 15 min at 4 °C.

The cell pellet was resuspended in PBS, pH 7.4, and lysed by sonication (12 cycles of 10-s sonication separated by 40-s intervals, 40% amplitude). Poly(ethyleneimine) was added to each lysed suspension to a final concentration of 1.25%, after which the soluble fraction was separated from the cell debris by 15 min of centrifugation at 4,000 × *g*. ELP-GFP proteins were then purified by phase transition triggered by sodium citrate, followed by centrifugation at 15,000 × *g* for 3 min to eliminate contaminant proteins that did not precipitate. Finally, native *E. coli* proteins were denatured at 75 °C, and removed by centrifugation. After three purification cycles, the ELP-GFP proteins to >95% purity as judged by Coomassie staining of SDS/PAGE gels.

### Protein Preparation and Functionalization.

When stated, pAF residues from ELP-GFP proteins were regenerated using ISAz, as previously described. In brief, diazotransfer reactions were performed for proteins at a concentration of 20 µM using 200 equivalents of ISAz in 10× PBS (1.4 M NaCl, 0.1 M phosphate, 0.03 M KCl) pH 7.2 at room temperature. After 72 h, reactions were stopped by exchanging the buffer to PBS (1×, pH7.4).

The ELP-GFP proteins were reacted with palmitic acid alkyne using copper(I)-catalyzed azide-alkyne Huisgen cycloaddition (click-chemistry). For this reaction, proteins were diluted to a final azide concentration of 30 µM, 35% DMSO, 0.16 mM palmitic acid alkyne, 0.1 mM CuSO_4_ and 0.5 mM THPTA (premixed for 30 min), 5 mM aminoguanidine hydrochloride, and 5 mM sodium ascorbate. The click-chemistry reaction was incubated for 1 h at room temperature under constant, gentle mixing. After the reaction, the protein was buffer exchanged to PBS (pH 7.4) using Amicon filters (10 kDa molecular weight cutoff [MWCO]).

Proteins for biodistribution studies were further labeled at primary amines with an Alexa Fluor 647 succinimidyl ester. Proteins were diluted to 0.1 mg/mL, and mixed with 5 µg/mL fluorophore in PBS for mild labeling. Excess dye was removed using Amicon filters (10 kDa MWCO).

Endotoxins were removed from all protein preparations used for animal experiments, using Pierce high-capacity endotoxin removal columns following the manufacturer’s protocol (Thermo Fisher Scientific, catalog # 88274). Prior to injection, endotoxin levels were confirmed to be under 0.1 endotoxin unit (EU) per injection using Gel-Clot LAL reagent with sensitivity of 0.06 EU/mL (Charles River, catalog #R12006).

### MS Characterization.

The purity at the target residue was determined by quantitative MS. The ELP-GFP proteins were buffer exchanged and diluted to 15 µM in digestion buffer (50 mM Tris, pH 8.0, and 0.5 mM CaCl_2_), and were digested with 1.5 µM thermolysin (Promega) for 6 h at 80 °C. The resulting ELP-peptides were quantified using standard curves based on synthetic peptides (*SI Appendix*, Fig. S2). High-resolution MS data were collected using an Agilent iFunnel 6550 quadrupole time-of-flight (TOF) MS with an electrospray ionization (ESI) source, coupled to an Agilent Infinity 1290 ultrahigh-performance liquid chromatography system with an Agilent Eclipse Plus C18 1.8 μm, 4.6 × 50-mm column. Solvents used were (solvent A) water 0.1% formic acid and (solvent B) CH_3_CN 0.1% formic acid. Mass spectra were gathered using Dual Agilent Jet Stream ESI in positive mode. The mass range was set from 110 to 1,700 *m/z* with a scan speed of three scans per second. The capillary and nozzle voltages were set to 5,500 and 2,000 V, respectively. The source parameters were set with a gas temperature of 280 °C and a flowrate of 11 liters/min, nebulizer at 40 psig, and sheath gas temperature at 350 °C at a flow of 11 liters/min. MS data were acquired with MassHunter Workstation Data Acquisition (version B.06.01, Agilent Technologies) and analyzed using MassHunterQualitative Analysis (version B.07.00, Agilent Technologies).

### Intact Mass by MALDI-TOF.

For MALDI-TOF analysis, 2 μL of the protein samples were mixed in a ratio of 1:1:1 with 2% trifluoroacetic acid solution and then with the matrix solution (375 μL of 20 mg/mL solution of 2,5-DHAP [2,5-dihydroxy acetophenone] in ethanol and 125 μL of 18 mg/mL of aqueous DAC [diammonium hydrogen citrate solution]) by pipetting, until crystallization of the mixture. Then 0.5 μL of the protein sample was loaded on MALDI steel target plate and analyzed after solvent evaporation.

MALDI-TOF MS spectra were acquired using an MALDI-TOF/TOF autoflex speed mass spectrometer (Bruker Daltonik), equipped with a smartbeam-II solid-state laser (modified Nd:YAG laser, λ = 355 nm), at the Ilse Katz Institute for Nanoscale Science and Technology (Ben-Gurion University of Negev, Beer-Sheva, Israel). The instrument was operated in positive ion, linear mode within a mass range from *m/z* 10 kDa to 50 kDa. Laser fluence were optimized for each sample. The laser was fired at a frequency of 1 kHz and spectra were accumulated in multiples of 500 laser shots, with 1,500 shots in total. Calibration was performed using protein calibration standard from Bruker. Spectrum analysis was performed by the Flexanalysis software.

### DLS Analysis.

Protein solubility and self-assembly was analyzed using a Zetasizer Nano ZS (Malvern Pananalytical). For each sample, 11 to 15 acquisitions (determined automatically by the instrument) were obtained at 25 °C for 10 μM protein solutions in PBS. Three separately prepared samples were analyzed, and the analysis for each sample was repeated three times. Populations comprising less than 1% of the total mass (by volume) were excluded from the analysis.

### SPR.

Binding assays were performed on a Biacore T200 instrument. HSA (Sigma, catalog #A3782) or albumin from mouse serum (Sigma, catalog #A3139) were immobilized by amine coupling to research grade CM5 chip (GE Healthcare, catalog #BR100530) from 20 µg/mL solutions in 10 mM acetate pH 5.0. High-density surfaces were created ranging from ∼1,300 to 12,800 RUs to minimize nonspecific binding of ELP-GFP derivatives. Binding was measured with 60-s association phase and 600-s dissociation phase with either no regeneration, or surfaces were regenerated with two 30-s pulses of 50 mM NaOH. ELP-GFP derivatives were injected in duplicates from twofold dilution series with at least six different concentrations ranging from ∼0.28 to 60 µM (depending on the polymer and its expected *K*_d_); PBS was used as running buffer. Data were doubly referenced against the signal collected on the reference cell and responses generated on the active cells during buffer injections. Data were analyzed using Evaluation software and fit into a steady-state affinity binding model. Each reported affinity is an average from four to eight independent measurements.

### Trx Activity Assay.

The activity of recombinant trx and trx fusion proteins were determined using the Proteostat thioredoxin-1 activity assay (Enzo). Trx catalyzed reduction of insulin and consequent aggregation of insulin in the presence of dithiothreitol (DTT) was monitored by a fluorescent dye. Trx activity was determined using a standard curve using a concentration range of bacterial trx, and the activity of samples were determined to be within the linear range of the assay. Fluorescence emission was monitored using a Biotek spectrophotometric plate reader.

### Mouse Models and In Vivo Experiments.

All experiments were performed in C57BL/6J mice in accordance with the guidelines of the Animal Care and Use Committee of Yale University. Recommendations from the *Guide for the Care and Use of Laboratory Animals* (49) were followed during these experiments.

The half-lives of ELP-GFP constructs were calculated from concentrations measured from blood samples collected over the course of a week. The experiments were initiated by injecting 120 µL of 10 µM ELP-GFP intravenously or subcutaneously. At indicated times, 2 µL blood was collected from a tail puncture, and diluted 1:25 in heparin tubes. The blood sample was vortexed briefly and cells were pelleted by centrifugation (2 min at 14,000 × *g*). The soluble fraction was collected and frozen at −20 °C until analysis. ELP-GFP concentrations of the samples were determined using a GFP ELISA Kit (Abcam, catalog #ab171581). The samples were diluted in PBS as needed, to ensure that the concentration fell within the quantifiable range of the standard curve.

To study the immunogenicity and biodistribution of ELP-GFP, 120 µL of 10 µM Alexa Fluor 647 labeled constructs were injected, and blood and organs were collected at indicated times. As positive control for an inflammatory response, 100 µg LPS was injected, and an injection of PBS was performed as negative control. Organs were imaged using Amersham Imager 600 RGB, and signal visualization and quantification were performed with FIJI (https://imagej.net/software/fiji/). For cytokine quantification, blood was allowed to coagulate, and serum was collected. Cytokines were quantified from the serum samples using the BD CBA Mouse Inflammation Kit (Fisher Scientific, catalog #BD 552364).

## Supplementary Material

Supplementary File

## Data Availability

Data supporting the findings of this work are available within the paper and its supporting information files. The strains and plasmid (sequences) have been deposited in GenBank or Addgene: Genetically recoded organism C321.deltaA (RRID: Addgene_48998), aminoacyl tRNA synthetase (RRID: Addgene_73545), ELP-GFP reporter (Genbank: KT996142). All other study data are included in the article and/or *SI Appendix*.
