# Supplementary material for: Tuning protein half-life in mouse using sequence-defined biopolymers functionalized with lipids
Source: Proc Natl Acad Sci U S A. 2022 Jan 18;119(4):e2103099119. doi: 10.1073/pnas.2103099119 (PMC8794819; doi:10.1073/pnas.2103099119)
Supplement: Supplementary File [file pnas.2103099119.sapp.pdf]

PNAS
www.pnas.org

Supplementary Information for

Tuning protein half-life in mouse using sequence-defined biopolymers functionalized with lipids

Koen Vanderschuren[1,2,8], Pol Arranz-Gibert[1,2,8], Minsoo Khang[3,8], Dagan Hadar[4,8], Alice Gaudin[3], Fan Yang[3], Ewa Folta-Stogniew[5], W. Mark Saltzman[3,6,7], Miriam Amiram[4,*], Farren J. Isaacs[1,2,3,*]
[1]Department of Molecular, Cellular & Developmental Biology, Yale University, New Haven, CT, 06520, USA.
[2]Systems Biology Institute, Yale University, West Haven, CT, 06516, USA.
[3]Department of Biomedical Engineering, Yale University, New Haven, CT, 06520, USA.
[4]Avram and Stella Goldstein-Goren Department of Biotechnology Engineering, Ben Gurion University of the Negev, Beer-Sheva, 84105, Israel.
[5]W.M. Keck Biotechnology Research Laboratory, Yale University School of Medicine, New Haven, CT 06511, USA.
[6]Department of Chemical & Environmental Engineering, Yale University, New Haven, CT, 06520, USA.
[7]Department of Cellular & Molecular Physiology, Yale University, New Haven, CT, 06511, USA.
[8]These authors contributed equally to this work.

[†]Correspondence to: Email: farren.isaacs@yale.edu (F.J.I.); mamiram@bgu.ac.il (M.A.).

This PDF file includes:

- Supplementary text
- Figures S1 to S14
- Tables S1 to S3
- SI References

Supplementary Information Text

Model of half-life extension.

In this model, the predicted half-life is determined by the composite clearance of ELP-GFP and ELP-GFP bound to albumin. As such, the half-life is determined by two factors. First, we propose that free and bound ELP-GFP have differential clearance rates, where the half-life of free ELP-GFP is experimentally determined from ELP(0FA)GFP, and the half-life of bound ELP-GFP follows that of albumin. Second, the ratio of free and bound ELP-GFP is determined by the binding affinity between ELP-GFP and albumin. Collectively, these factors can be described by four ordinary differential equations:

$$r_1 = [ELP] \times [Albumin] \times k_a$$

$$r_2 = [Complex] \times k_d$$

$$r_3 = [ELP] \times \frac{\ln(2)}{\tau_{ELP}}$$

$$r_4 = [Complex] \times \frac{\ln(2)}{\tau_{Albumin}}$$

where k_a is the association constant for binding between albumin and ELP-GFP, and k_d is the dissociation constant. The reaction r_1 represent the binding of ELP-GFP to albumin and is dependent on the concentrations of both. The binding is reversible, and r_2 expresses the dissociation of the complex. Furthermore, τ_{ELP} and $\tau_{Albumin}$ are the half-lives of unbound ELP(0FA)GFP and albumin, respectively. The reactions r_3 and r_4 describe the exponential decay of unbound ELP(0FA)-GFP and albumin, where τ_{ELP} and $\tau_{Albumin}$ are their respective half-lives.

The equations in this dynamical system allow calculation of the change of the three variables [ELP], [Albumin] and [Complex]:

$$\frac{d[ELP]}{dt} = -r_1 + r_2 - r_3$$

$$\frac{d[Albumin]}{dt} = -r_1 + r_2 + r_4$$

$$\frac{d[Complex]}{dt} = +r_1 - r_2 - r_4$$

In this system, the concentration of total albumin is kept constant by reintroducing unbound albumin equal to the amount of bound albumin that was degraded.

Starting concentrations for [Albumin], [ELP] and [Complex] were set to 250uM, 10uM and 0uM, respectively. The system of differential equations was solved in python, using scipy.integrate.odeint, at 1s intervals for 144h. The half-life was calculated using linear regression on the log transformed $[ELP]_{total}$ (*i.e.,* bound and unbound) after complex formation has reached equilibrium.

Binomial distribution of protein functionalization.

For the following predictions, we assume that each ELP(pAzF) unit is independent, and the probability of tyrosine misincorporation and pAzF reduction are unaffected by their position. As shown in ***SI Appendix*, Fig. S4**, the probability of having an unreduced pAzF residue is determined by the probability of tyrosine misincorporation, p_1, and the probability of pAzF reduction, p_2.

The number of pAzF residues per protein will follow a binomial distribution, which can be described as

$$\mathbb{P}\{X = k\} = \binom{n}{k} p^k (1-p)^{n-k} = C_k^n p^k (1-p)^{n-k} = \frac{n!}{k!(n-k)!} p^k (1-p)^{n-k}.$$

Here, n represents the number of UAG codons per protein, and k is the number of those positions that contain unreduced pAzF. Finally, p is the probability that a UAG codon results in an unreduced pAzF residue. As such, $p = p_1 \times p_2$.

The probabilities p_1 and p_2 were derived from empirical data in this work (**Fig. 2B** and ***SI Appendix*, Fig. S3**). The resulting distribution was compared to intact mass spectrometry data of impure ELP(10FA)GFP, and the correlation is reported.

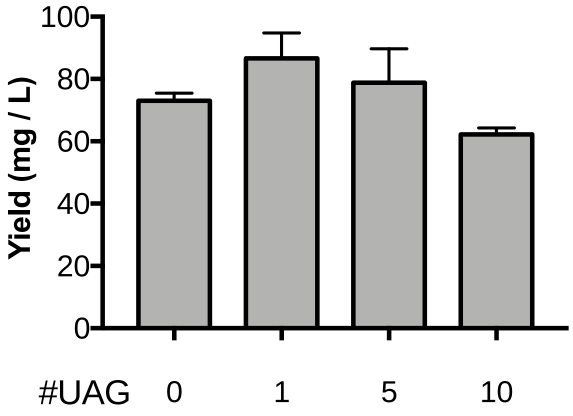

Fig. S1. Expression yields for ELP-GFP with multiple pAzF residues in the GRO. Yields in mg/L for ELP-GFP expression with 0, 1, 5, or 10 UAG codons. Yields were determined after cell lysis, and before purification of the ELP-GFP to minimize experimental biases resulting from purifications (n=3, error bars: mean ± s.d.).

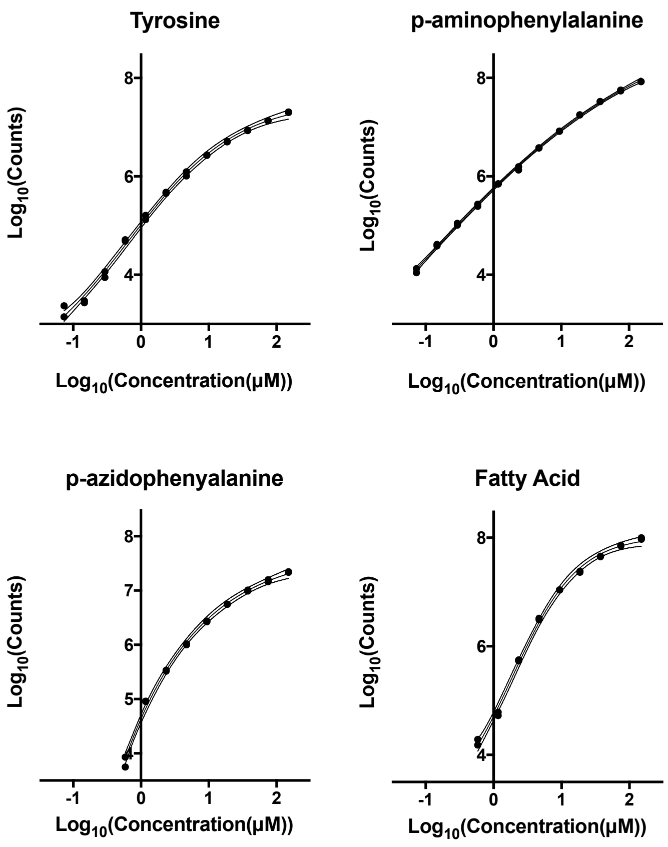

Fig. S2. Calibration curves for quantitative MS analysis of ELP-peptide species. Ion counts in replicate measured for 2-fold dilution series, starting at 150μM. Interpolation using a four-parameter logistic curve is shown (solid black line; dotted lines indicate 95% confidence interval). Concentrations of ELP-peptides in samples were computed from the resulting fit, sample ion counts fell within the range quantified by the standard curves.

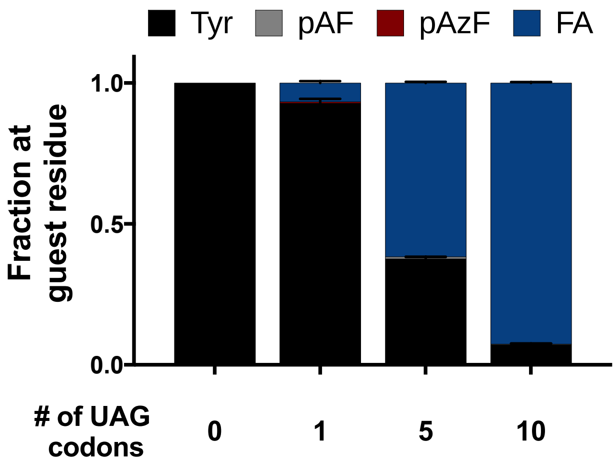

Fig. S3. Analysis of bulk ELP peptides after ISAz treatment and click-chemistry. Analysis of ELP-ion counts after ISAz and click chemistry, including ELP(Tyr) peptides. Each construct contains 10 ELP units that were quantified. At UAG codons, a pAzF residue was encoded, whereas other ELP units had tyrosine encoded at the target residue. The ELP(Tyr) counts are inversely correlated with the number UAG codons (n=3, error bars: mean ± s.d.).

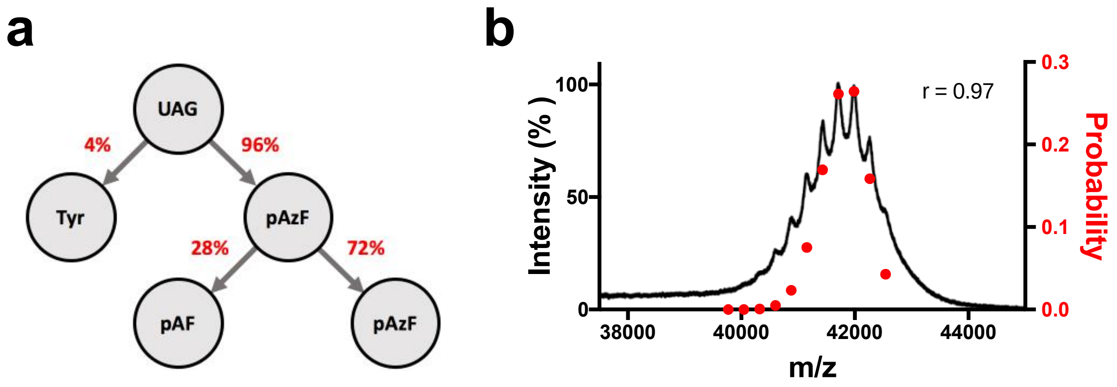

Fig. S4. Intact MS peak intensities and binomial distribution correlate strongly. (*A*) The distribution of FAs per protein is dependent on the availability of pAzF. We assume a 4% chance that any UAG codon is decoded by tyrosine, instead of pAzF. Further, assuming a 28% reduction of pAzF to pAF. The mathematical description is presented in the Supplementary Text. (*B*) Intact MS peak intensities of untreated ELP(10FA)GFP are compared to the probability distribution for 0-10FA per protein based on the binomial distribution specified in A.

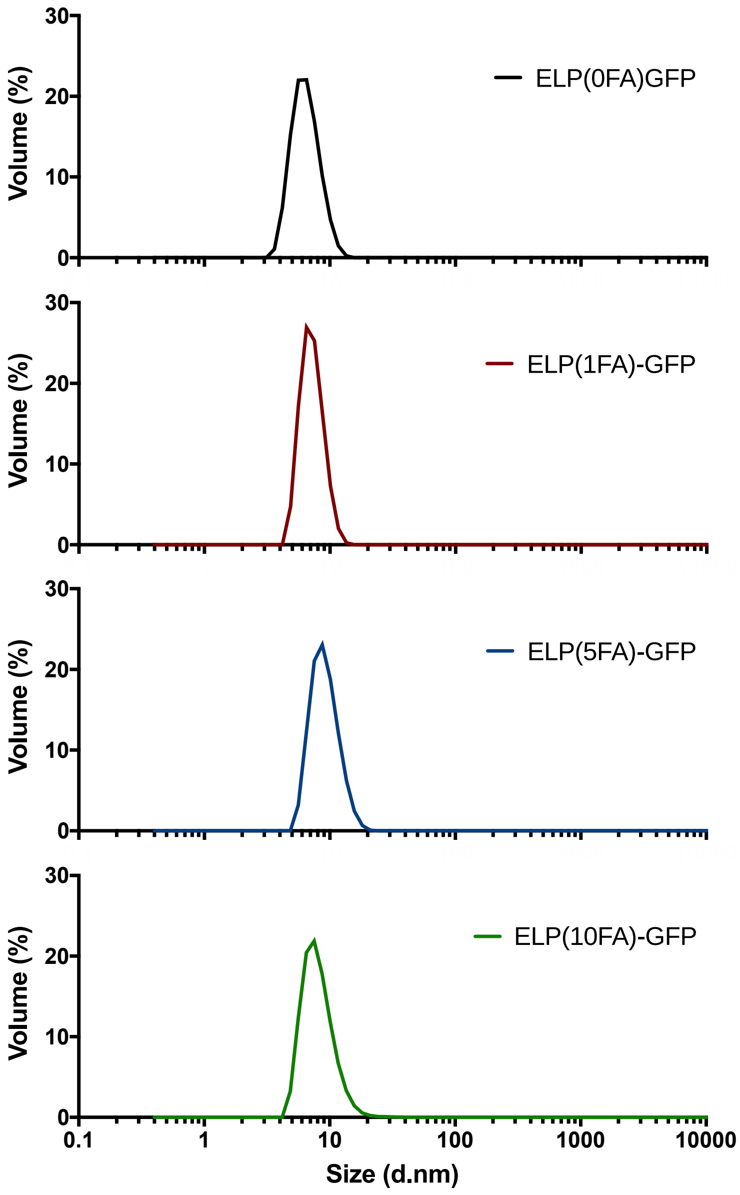

Fig. S5. Analysis of ELP(nFA)GFP size distribution by DLS. Samples with 1, 5, or 10 fatty acids per protein were ISAz treated before conjugation of the fatty acids. All samples had a similar size distribution, and the size distribution suggests minimal aggregation at a sample concentration of 10μM in PBS (pH7.4). The results are representative of 5 or more measurements per construct.

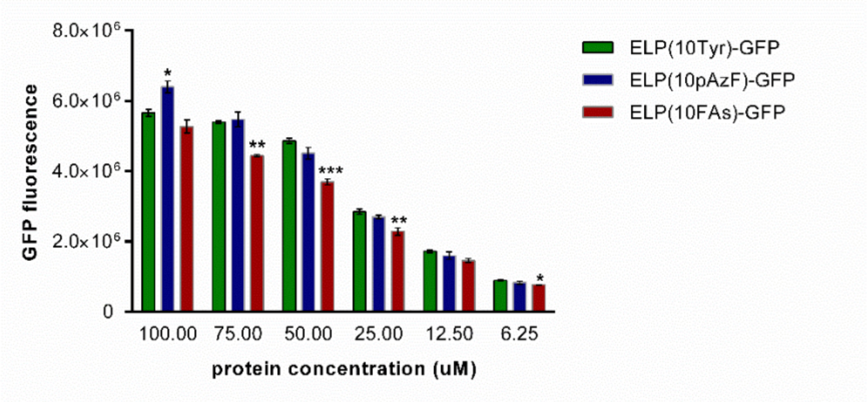

Fig. S6. Evaluation of the effect of FA conjugation on GFP fluorescence in fusion proteins ELP(10tyr)-GFP, ELP(10pAzF)-GFP and ELP(10FA)-GFP, which were examined at protein quantities ranging from 6.25-100 uM. (n=3, error bars: mean ± s.e.m.).*p<0.05, **p<0.01, ***p<0.001

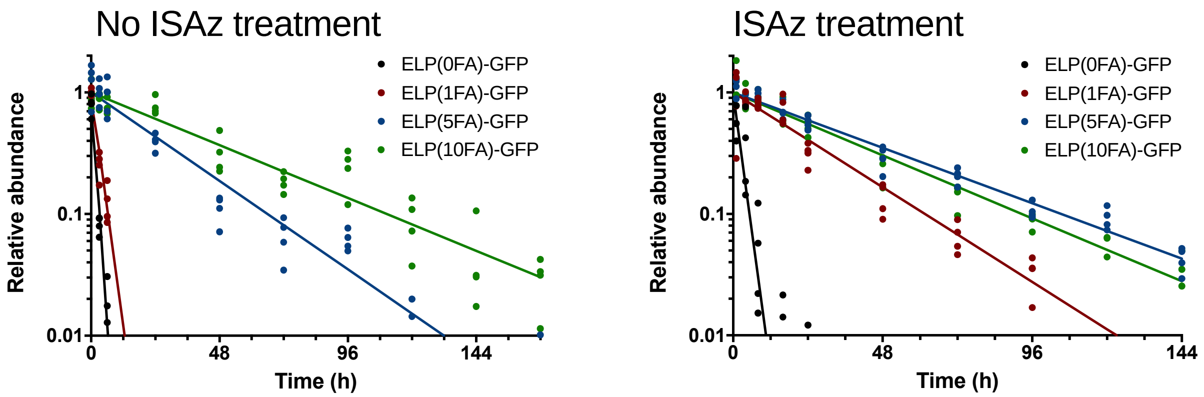

Fig. S7. Time course of ELP(FA)GFP in blood serum following intravenous (IV) administration. Left and right panels show measurements for ELP(FA)GFP without and with ISAz treatment, respectively. • represent individual measurements, normalized to the intercept (Time = 0 h) calculated for each dataset. The solid lines show the predicted abundance over time for each ELP(FA)GFP biopolymer based on the average half-life shown in Fig. 3A. The ELP(0FA)GFP biopolymer in both datasets did not undergo ISAz treatment or click-chemistry. n=4 for each biopolymer.

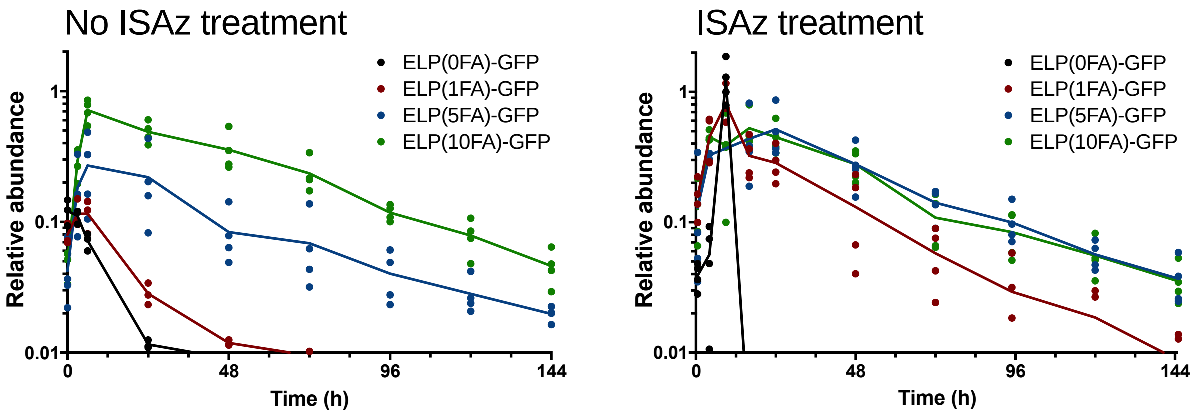

Fig. S8. Time course of ELP(FA)GFP in blood serum following subcutaneous (SC) administration. Left and right panels show measurements for ELP(FA)GFP without and with ISAz treatment, respectively. • represents individual measurements, normalized to the intercept (Time = 0 h) calculated for each dataset based on the data section following a logarithmic decrease. The solid line shows the average abundance for each ELP(FA)GFP biopolymer over time. The ELP(0FA)GFP biopolymer in both datasets did not undergo ISAz treatment or click-chemistry. n=4 for each biopolymer.

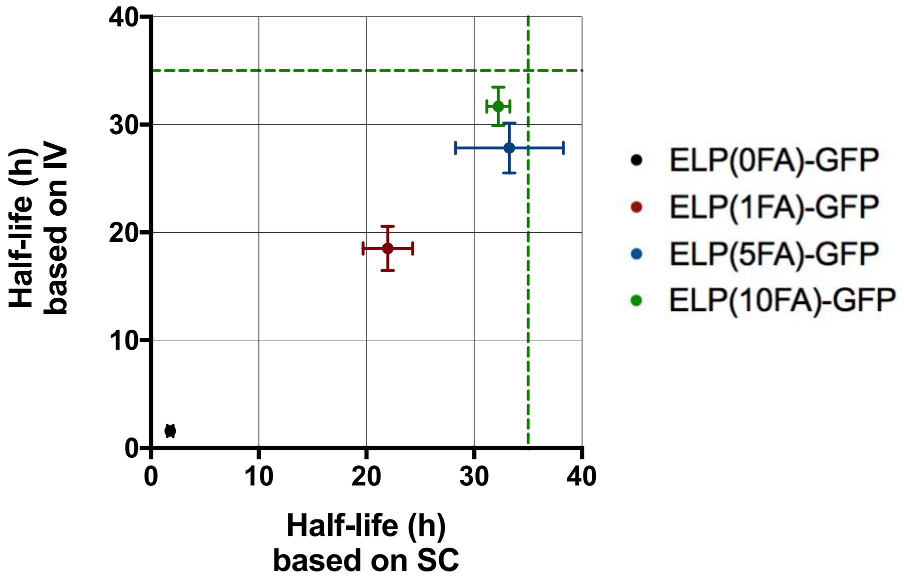

Fig. S9. Correlation between SC and IV half-lives. Comparison of half-life estimations derived from the IV injections and the logarithmic decrease of the SC injections of ISAz-treated ELP(nFA)-GFP proteins. The green, dashed lines indicate the 35 hours half-life of mouse serum albumin (n=4, error bars: mean ± s.d.).

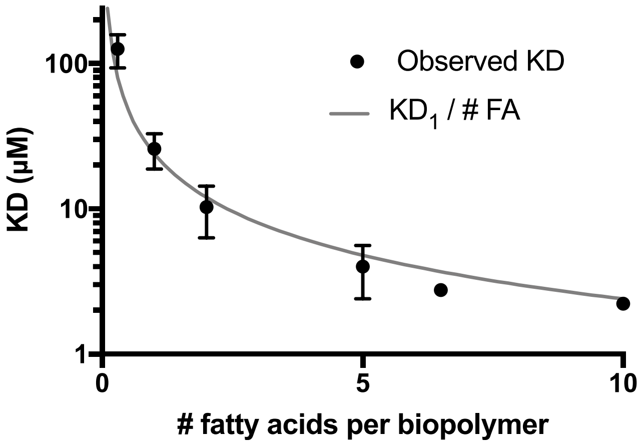

Fig. S10. Inverse correlation between KD value and number of fatty acids per protein. The average number of fatty acids per protein were inferred from quantitative MS characterization of the target residue (main text, figure 2), and KD values were determined by SPR (main text, table 1). (n=4-8, error bars: mean ± s.d.).

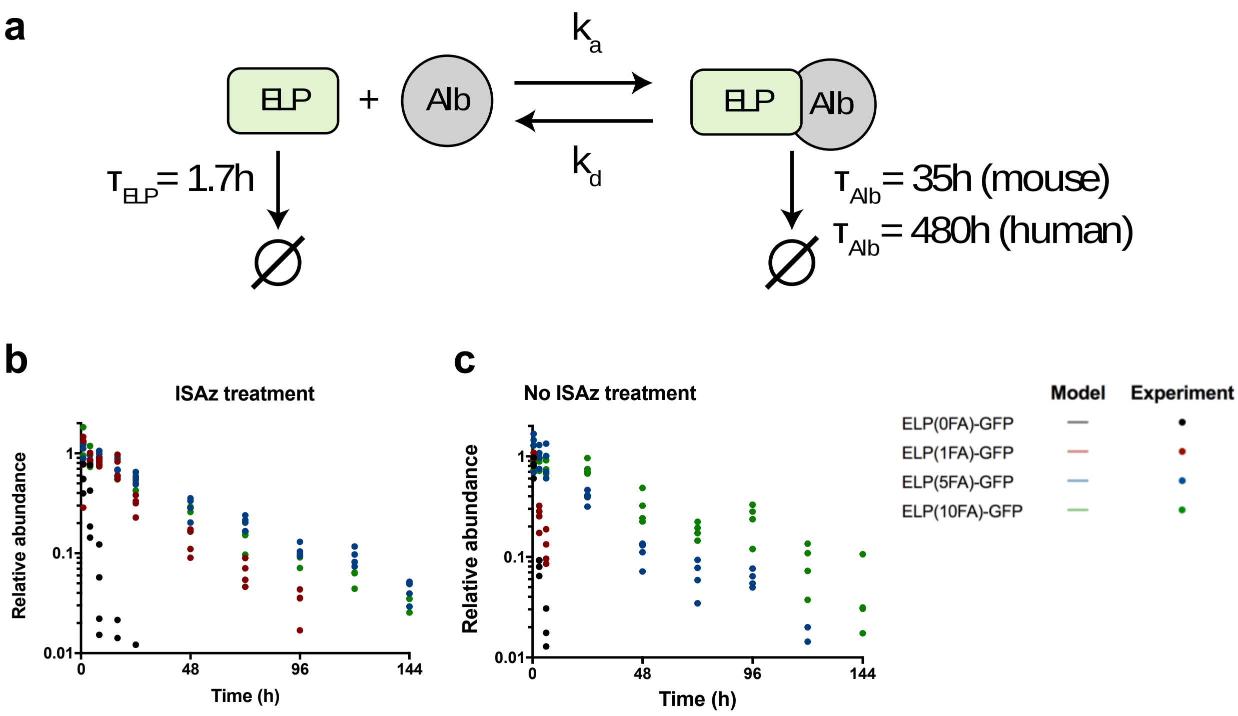

Fig. S11. Kinetic model of ELP clearance. (*A*) Model for computational prediction of half-life as a function of K_D (as described in the **Supplementary Text**). The half-life values are based on empirical data for unbound ELP-GFP obtained in this work and on reference value from (1) for albumin half-life. (*B* and *C*). Comparison of model predictions to measured ELP-GFP abundance over time. ELISA measurements are shown as •, model predictions are shown in solid lines. Comparison are made for ISAz treated constructs in (*B*), and untreated constructs in (*C*).

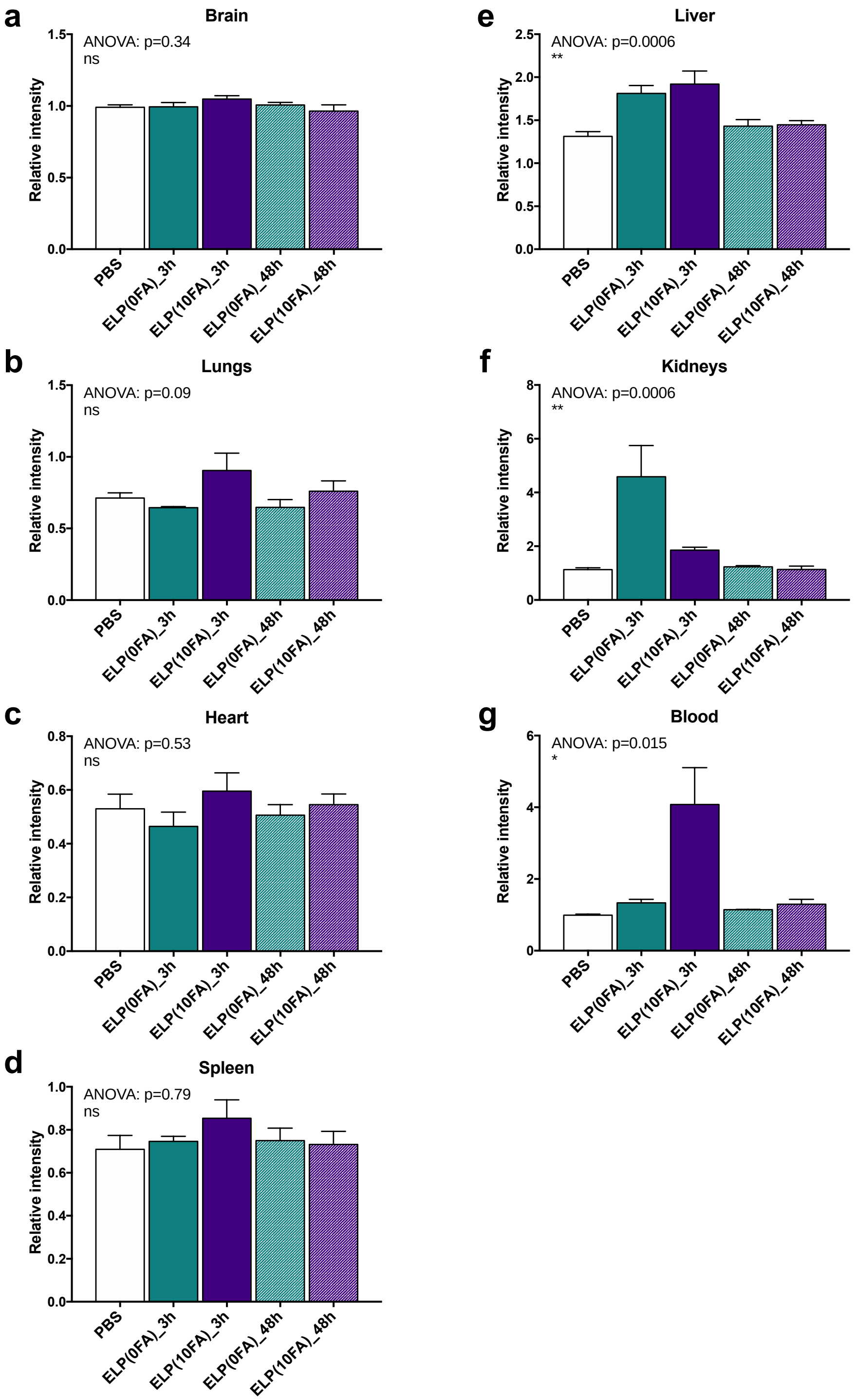

Fig. S12. Quantification of Alexa Fluor 647 signal intensity in mouse organs normalized to signal intensity for brain. For each organ set, a one-way ANOVA test was calculated to identify statistically significant differences in mean signal intensity between treatment groups. Results are shown for brain (*A*), lungs (*B*), heart (*C*), spleen (*D*), liver (*E*), kidney (*F*), and blood (*G*) (n=4, error bars: mean ± s.e.m.).

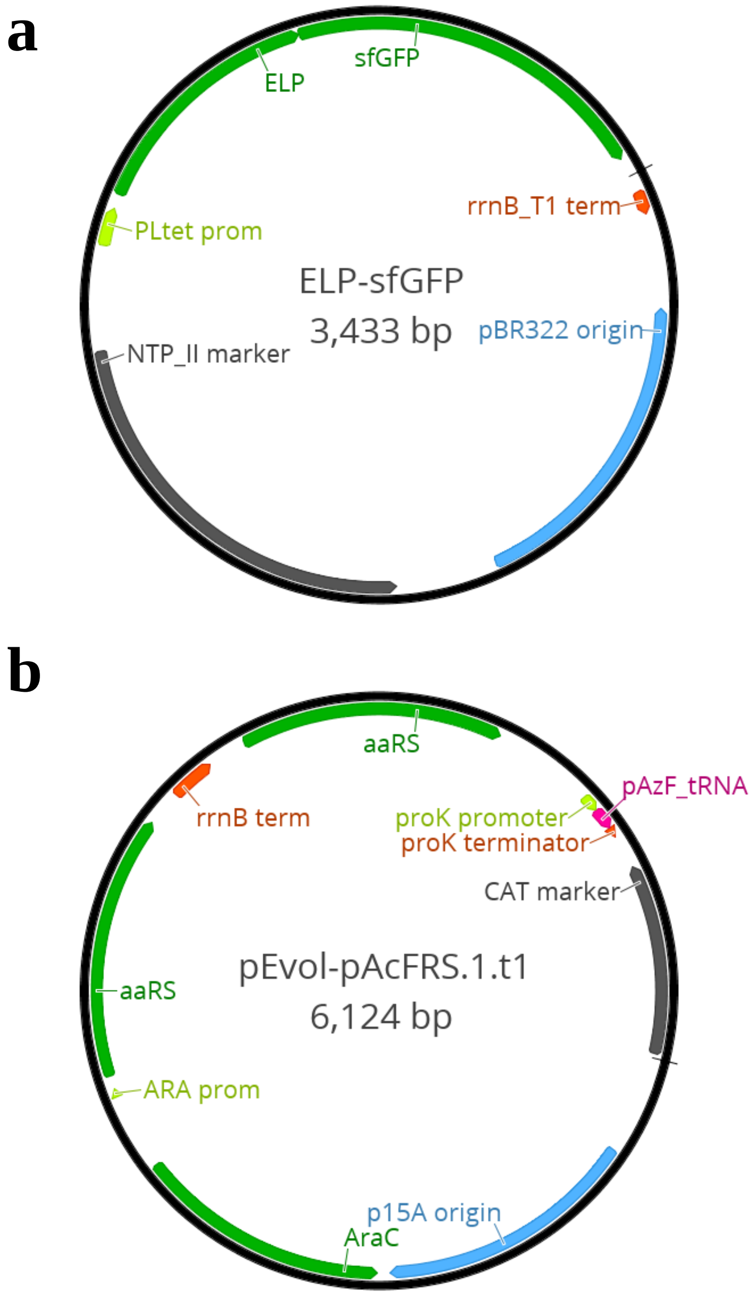

Fig. S13. Plasmid maps for reporter and OTS used in this study. (*A*) Plasmid map for ELP-GFP expression construct. (*B*) Plasmids map for the pAcFRS.1.t1 OTS used in this study. Both were previously described in (2). Green, grey, and blue sequences indicate expressed genes, resistance markers and origins of replication, respectively. Terminators are shown in orange, the OTS tRNA is shown in pink and promoters are displayed in light green.

Table S1. Pharmacokinetic characterization of ELP(nFA)-GFP constructs after intravenous administration. Values were derived from data presented in **Fig. S6**.

**Construct**	**ISAz treated**	**Half-life (h)**	**AUC (mg·h/L)**	**Distribution Volume (mL)**	**Clearance (mL/h)**
ELP(0FA)GFP	No	1.6	3	19.2	15.7
ELP(1FA)GFP	No	1.9	7	18.1	6.7
ELP(5FA)GFP	No	19.6	69	22.4	0.72
ELP(10FA)GFP	No	33.3	165	11.9	0.30
ELP(1FA)GFP	Yes	18.5	66	18.7	0.75
ELP(5FA)GFP	Yes	31.7	136	16.4	0.36
ELP(10FA)GFP	Yes	27.9	132	15.3	0.38

Table S2. Amino acid sequence of reporter proteins used in this study.

Protein name	Amino acid sequence
**GFP-WT** (superfolder)	SKGEELFTGVVPILVELDGDVNGHKFSVRGEGEGDATNGKLTLKFICTTGKLPVPWPTLVTTLTYGVQCFSRYPDHMKRHDFFKSAMPEGYVQERTISFKDDGTYKTRAEVKFEGDTLVNRIELKGIDFKEDGNILGHKLEYNFNSHNVYITADKQKNGIKANFKIRHNVEDGSVQLADHYQQNTPIGDGPVLLPDNHYLSTQSVLSKDPNEKRDHMVLLEFVTAAGITHGMDELYKGS
**ELP(0UAG) GFP**	MSKGPG(VPGGGVPGAGVPGYG)$_{10}$ PGGGG – (GFP-WT)
**ELP(1UAG) GFP**	MSKGPG(VPGGGVPGAGVPG(pAzF)G)$_{1}$(VPGGGVPGAGVPGYG)$_{9}$ PGGGG–(GFP-WT)
**ELP(5UAG) GFP**	MSKGPG(VPGGGVPGAGVPG(pAzF)GVPGGGVPGAGVPGYG)$_{5}$ PGGGG – (GFP-WT)
**ELP(10UAG) GFP**	MSKGPG(VPGGGVPGAGVPG(pAzF)G)$_{10}$ PGGGG – (GFP-WT)
**Trx**	MSDKIIHLTDDSFDTDVLKADGAILVDFWAEWCGPCKMIAPILDEIADEYQGKLTVAKLNIDQNPGTAPKYGIRGIPTLLLFKNGEVAATKVGALSKGQLKEFLDANLAGSGSGHMHHHHHHSSGAGP
**Trx-ELP(10UAG)**	SDKIIHLTDDSFDTDVLKADGAILVDFWAEWCGPCKMIAPILDEIADEYQGKLTVAKLNIDQNPGTAPKYGIRGIPTLLLFKNGEVAATKVGALSKGQLKEFLDANLAGSGSGHMHHHHHHSSGAGPG(VPGGGVPGAGVPG(pAzF)G)$_{10}$PGGG

Table S3. DNA sequence of ELP(0AUG)-GFP used in this study. Variable codons are highlighted in yellow.

ATGAGCAAAGGTCCCGGGGTTCCGGGTGGCGGCGTGCCGGGCGCAGGTGTTCCGGGTTAT
GGTGTGCCGGGCGGCGGTGTCCCGGGTGCTGGTGTGCCGGGCTACGGTGTCCCGGGTGG
CGGTGTTCCGGGCGCTGGTGTCCCGGGTTATGGTGTCCCGGGTGGCGGTGTTCCGGGTGC
AGGCGTTCCGGGTTACGGCGTGCCGGGCGGCGGTGTTCCGGGTGCTGGTGTGCCGGGCT
ATGGTGTCCCGGGTGGCGGTGTGCCGGGCGCAGGTGTCCCGGGTTACGGTGTTCCGGGC
GGCGGTGTCCCGGGTGCAGGTGTGCCGGGCTATGGTGTTCCGGGTGGCGGGGTGCCGGG
CGCTGGTGTTCCGGGTTATGGTGTGCCGGGCGGCGGTGTCCCGGGTGCAGGTGTGCCGG
GCTACGGTGTCCCGGGTGGCGGTGTTCCGGGCGCAGGTGTCCCGGGTTATGGGCCCGGC
GGTGGGGGCAGCAAGGGCGAAGAACTGTTTACGGGCGTGGTGCCGATTCTGGTGGAACTG
GATGGTGATGTCAATGGTCACAAATTCAGCGTGCGCGGCGAAGGTGAAGGCGATGCAACCA
ATGGTAAACTGACGCTGAAGTTTATTTGCACCACGGGTAAACTGCCGGTTCCGTGGCCGAC
CCTGGTCACCACGCTGACGTATGGTGTTCAGTGTTTCAGTCGTTACCCGGATCACATGAAAC
GCCACGACTTTTTCAAGTCCGCGATGCCGGAAGGTTATGTCCAAGAACGTACCATCTCATTT
AAAGATGACGGCACCTACAAAACGCGCGCCGAAGTGAAATTCGAAGGTGATACGCTGGTTA
ACCGTATTGAACTGAAAGGCATCGATTTTAAGGAAGACGGTAATATTCTGGGCCATAAACTG
GAATATAACTTCAATTCGCACAACGTGTACATCACCGCAGATAAGCAGAAGAACGGTATCAA
GGCTAACTTCAAGATCCGCCATAATGTGGAAGATGGCAGCGTTCAACTGGCCGACCACTAT
CAGCAAAACACCCCGATTGGTGATGGCCCGGTCCTGCTGCCGGACAATCATTACCTGAGCA
CGCAGTCTGTGCTGAGTAAAGATCCGAACGAAAAGCGTGACCACATGGTCCTGCTGGAATT
CGTGACCGCGGCCGGCATCACGCACGGTATGGACGAACTGTATAAAGGCTCATAA

SI References

1. C. Chaudhury, et al., The major histocompatibility complex-related Fc receptor for IgG (FcRn) binds albumin and prolongs its lifespan. *J. Exp. Med.* **197**, 315-322 (2003).
2. M. Amiram, et al., Evolution of translation machinery in recoded bacteria enables multi-site incorporation of nonstandard amino acids. *Nat. Biotechnol.* **33**, 1272-1279 (2015).
